# A real-time contouring feedback tool for consensus-based contour training

**DOI:** 10.3389/fonc.2023.1204323

**Published:** 2023-09-13

**Authors:** Christopher L. Nelson, Callistus Nguyen, Raymond Fang, Laurence E. Court, Carlos E. Cardenas, Dong Joo Rhee, Tucker J. Netherton, Raymond P. Mumme, Skylar Gay, Casey Gay, Barbara Marquez, Mohammad D. El Basha, Yao Zhao, Mary Gronberg, Soleil Hernandez, Kelly A. Nealon, Mary K. Martel, Jinzhong Yang

**Affiliations:** Department of Radiation Physics, The University of Texas MD Anderson Cancer Center, Houston, TX, United States

**Keywords:** contour training, contour variability, consensus contouring, radiotherapy planning, localized signed surface distance

## Abstract

**Purpose:**

Variability in contouring structures of interest for radiotherapy continues to be challenging. Although training can reduce such variability, having radiation oncologists provide feedback can be impractical. We developed a contour training tool to provide real-time feedback to trainees, thereby reducing variability in contouring.

**Methods:**

We developed a novel metric termed localized signed square distance (LSSD) to provide feedback to the trainee on how their contour compares with a reference contour, which is generated real-time by combining trainee contour and multiple expert radiation oncologist contours. Nine trainees performed contour training by using six randomly assigned training cases that included one test case of the heart and left ventricle (LV). The test case was repeated 30 days later to assess retention. The distribution of LSSD maps of the initial contour for the training cases was combined and compared with the distribution of LSSD maps of the final contours for all training cases. The difference in standard deviations from the initial to final LSSD maps, ΔLSSD, was computed both on a per-case basis and for the entire group.

**Results:**

For every training case, statistically significant ΔLSSD were observed for both the heart and LV. When all initial and final LSSD maps were aggregated for the training cases, before training, the mean LSSD ([range], standard deviation) was –0.8 mm ([–37.9, 34.9], 4.2) and 0.3 mm ([–25.1, 32.7], 4.8) for heart and LV, respectively. These were reduced to –0.1 mm ([–16.2, 7.3], 0.8) and 0.1 mm ([–6.6, 8.3], 0.7) for the final LSSD maps during the contour training sessions. For the retention case, the initial and final LSSD maps of the retention case were aggregated and were –1.5 mm ([–22.9, 19.9], 3.4) and –0.2 mm ([–4.5, 1.5], 0.7) for the heart and 1.8 mm ([–16.7, 34.5], 5.1) and 0.2 mm ([-3.9, 1.6],0.7) for the LV.

**Conclusions:**

A tool that uses real-time contouring feedback was developed and successfully used for contour training of nine trainees. In all cases, the utility was able to guide the trainee and ultimately reduce the variability of the trainee’s contouring.

## 1 Introduction

Radiotherapy represents a balance between local tumor control and minimizing toxicity to normal tissues. Treatment plans are designed so that the prescription dose is delivered to the target while minimizing dose to nearby organs at risk (1, 2). Radiotherapy treatment planning begins with accurate delineation of both the target volume and organs at risk. Previous studies have shown substantial variations exist in the contouring process, including both intra-observer and inter-observer variations (3–8). These variations usually result from differences in training on how to generate contours and can be significantly influenced by the image quality of the contouring dataset. This is particularly true when contouring organs with low contrast relative to surrounding tissues. Previous studies that analyzed inter-observer variability in contouring suggested the need for consensus training in contouring (4). Large clinical trials also called for consistent contouring across different institutions to produce meaningful outcomes in analyses of treatment-related toxicity (9).

Traditional contour training usually involves experienced attending radiation oncologists providing feedback to trainees via interactive teaching tools. This approach requires a significant commitment from physicians to their clinical workload; the feedback provided is often delayed, sometimes by several days. Such delays may reduce the effectiveness of contouring training. This interactive training approach is also more subjective than objective. Various online contouring training tools have been developed to address these shortcomings [e.g., eContour (10) and EduCase (11)], but most of these tools do not give meaningful feedback on how well a trainee is contouring and often rely on assumed “gold standard” contours. Errors in “gold standard” contours could introduce bias, especially for low-contrast organ contours, and may not be helpful for trainees to improve their contouring skills. Although quantitative metrics are available in these tools to characterize contouring performance, they usually do not include any spatial or shape information of the organ being evaluated. As a result, these contour training tools cannot tell the trainee specifically where to adjust the contour to improve consistency.

In this study, we developed a software tool for consensus contouring training that provides real-time feedback on contouring performance without the presence of radiation oncologist staff who traditionally fill this role. We developed a new quantitative metric containing spatial information for analysis of inter-observer variability that can guide the trainee to the specific location where contours need to be adjusted. This new metric enables real-time feedback on contouring performance to the trainee so that they can continuously practice contouring without interruption. We propose that our tool can improve training efficiency by providing real-time feedback without the need for experienced radiation oncologists to be present. Not only does this benefit modern radiation oncology clinics, where radiation oncologists’ time is at a premium, but this tool can also be useful in low- to middle-income countries, which often have a great need for radiation oncology staff trained in contouring but resources are limited (12–14).

## 2 Methods

### 2.1 Overview of contour training tool

A contour training software utility was developed to provide real-time feedback on contouring to the user (referred to here as the trainee). The utility serves as a full contouring package, as it includes contouring tools commonly found in commercial treatment planning systems. The utility can also provide real-time contouring feedback to the trainee while they contour a region of interest or a specific organ. This real-time feedback can guide the trainee to specific spatial locations where contours need adjustment to improve consistency. This utility stores numerous contours from expert radiation oncologists that are used to quantify the consensus-contouring performance of the trainee. This real-time contouring feedback is expected to improve the trainees’ skills in consensus contouring.

### 2.2 Localized signed surface distance (LSSD)

**Figure 1:**
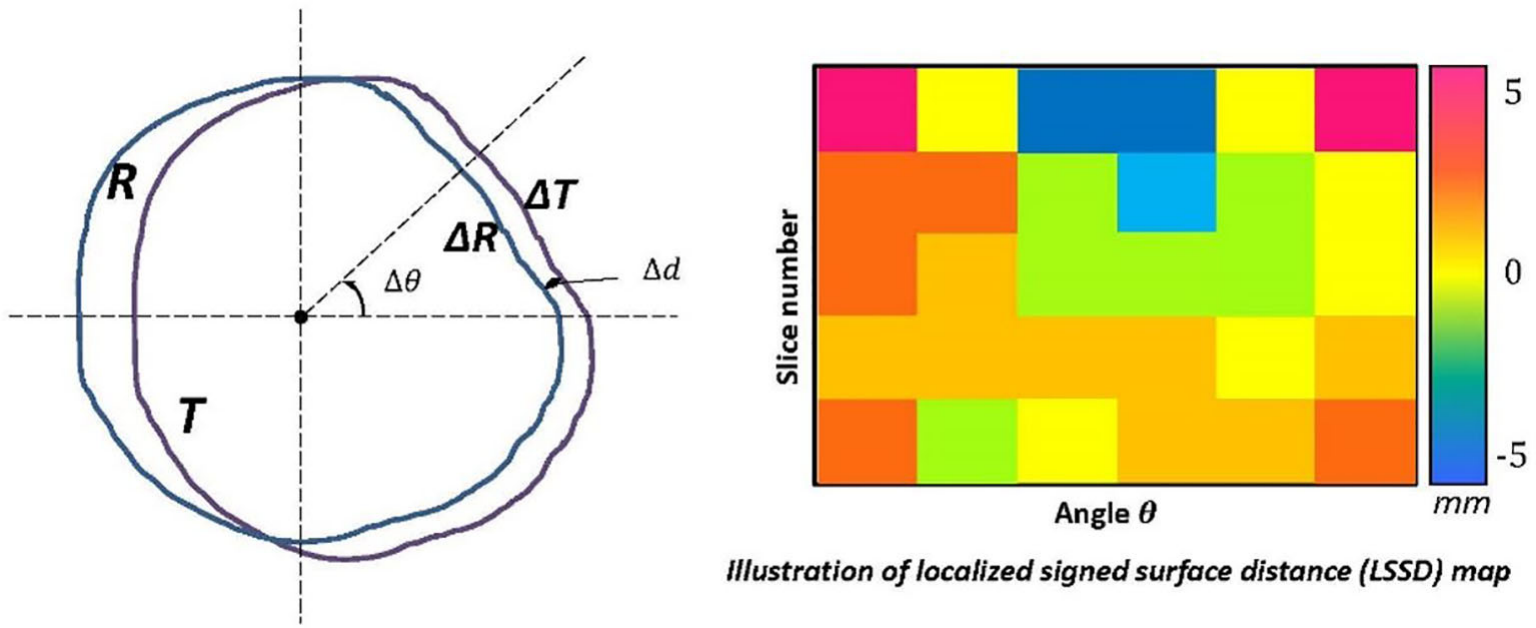
Graphic illustration of the LSSD algorithm. Illustration of the quantitative metric with spatial information, the localized signed surface distance (LSSD). The entire space in one slice is separated into small sectors, with each sector spanned by an angle of Δθ with the origin at the geometric center of reference contour (R). In each sector, the mean distance Δd between the piece of reference contour, ΔR , and the piece of trainee contour (*T*), ΔT , is calculated. The sensitivity and specificity of trainee contours are calculated by using the volumes enclosed by ΔR and ΔT , and they are used to determine a positive or negative distance for this sector. The distance information from all sectors and slices is then transformed to an LSSD color map to demonstrate variations in contouring.


A new quantitative metric was developed for real-time contouring feedback called localized signed surface distance (LSSD), which is based on mean surface distance. Specifically, for one structure, the disagreement between the trainee contour (T) and the reference contour (R) is examined within each slice. In one slice, first the geometric center of the reference contour is determined. Then the entire space in the slice is divided into $N=\left[\frac{2\pi }{\text{Δ}\theta }\right]$ sectors, with each sector spanned by an angle of Δθ , as shown in **Figure 1**. In each sector, the mean distance |Δd| between the section of reference contour, ΔR , and the section of manual contour, ΔT , is calculated as:

(1) $$\begin{array}{c} \left|\text{Δ}d\right|=\frac{\text{Δ}d_{RT}+\text{Δ}d_{TR}}{2}\;\text{with}\;\text{Δ}d_{RT}\\ =\frac{1}{\left|\text{Δ}R\right|}\sum_{r\in \text{Δ}R}\underset{t\in \text{Δ}T}{\min}d\left(r,t\right)\;\text{and}\;\text{Δ}d_{TR}=\frac{1}{\left|\text{Δ}T\right|}\sum_{t\in \text{Δ}T}\underset{r\in \text{Δ}R}{\min}d\left(t,r\right). \end{array}$$

The volumes enclosed by ΔR and ΔT in the sector are then used to calculate the sensitivity (*P*) and specificity (*Q*) for the manual contour with regard to the reference contour as:


(2) $$P=\frac{V_{\text{Δ}R}\cap^{}V_{\text{Δ}T}}{V_{\text{Δ}R}};Q=\frac{V_{\text{Δ}R}\cap^{}V_{\text{Δ}T}}{V_{\text{Δ}T}}.$$

The sensitivity and specificity are used to determine a positive or negative distance for this sector:


(3) If P≥Q, Δd=|Δd|;   If P<Q, Δd=−|Δd|.

A positive distance suggests that the trainee’s contour is larger than the reference contour or that the observer tends to draw contours that are larger than they need to be at this specific location. On the other hand, a negative distance suggests that the trainee’s contour is smaller than the reference contour or that the observer tends to draw contours that are smaller than what they should be at this specific location. Our LSSD definition is similar to the distance deviation measure proposed by Rogelj et al. (15) and radial distance proposed by Sebastien et al. (16); however, our LSSD is a signed distance to indicate over- or under- contoured. The distance information of all sectors and slices is transformed to an LSSD map. The signed distance is colorized to easily identify the disagreement. In the common head-first supine setup, the angle θ of 0°, 90°, 180°, and 270° represents left, anterior, right, and posterior locations, respectively. After the trainee completes contouring a structure, the LSSD map is updated, which provides the real-time feedback on contouring performance to the trainee. With the LSSD map, the trainee can quickly identify the spatial locations of inconsistent contours. Also, an appropriate threshold can be applied to the LSSD map to emphasize large variations.

### 2.3 Reference expert contours

Six training cases involving contours of the heart and left ventricle (LV) were used to validate the effectiveness of the training tool. These training cases were adopted from a set of atlases that were developed for an automatic multi-atlas contouring system (17). The heart and LV are important structures to spare dose for cardiac toxicity control during radiotherapy planning (18–20). Studies have found that inconsistent contouring of these structures has greatly compromise the toxicity control (9). In this study, the heart was chosen to represent a relatively easy structure for consistent contouring while the LV was chosen to represent a relatively difficult case because of its low contrast to other heart chambers (21). For each training case, eight experts specializing in either thoracic radiation oncology or lymphoma radiation oncology delineated the heart and left ventricle individually according to the RTOG (Radiation Therapy Oncology Group) 1106 organ-at-risk contouring guideline (22) and a published cardiac atlas consensus contouring guideline (23). The contours were drawn on non-contrast CT images in the Pinnacle treatment planning system (Philips Medical Systems, Fitchburg, WI), with corresponding contrast CT images rigidly fused to constitute the reference. The manual contours of the eight experts and the CT images for all 6 cases were exported from the treatment planning system and imported into the stand-alone contour-training tool. These expert contours were used to generate the reference contours for LSSD map computation, as described in the next section.

### 2.4 Contour training software interface

**Figure 2:**
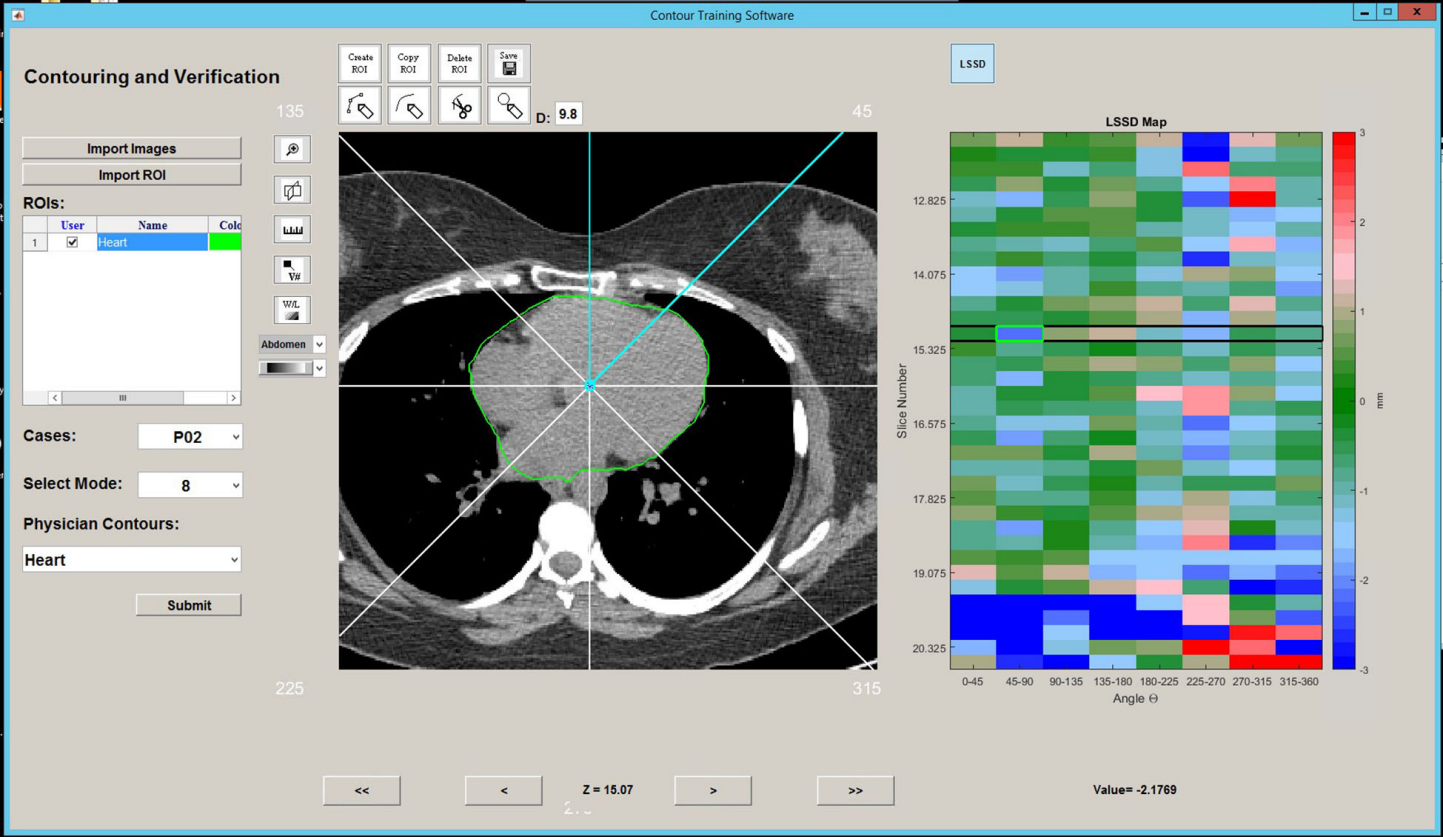
Contouring interface with LSSD map. An example of the contour training interface showing the contouring panel (left) and the interactive LSSD map (right).

Within the training software interface, the trainee is first prompted to select a training case and training structure (heart or LV). Once the training case is loaded into the software interface, only the CT image is shown. The trainee first creates and contours the entire region of interest on the CT scan. Behind the interface, the software creates a reference contour by fusing the trainee contour with those of the eight experts by using the simultaneous truth and performance level estimation (STAPLE) algorithm (24, 25). The STAPLE algorithm is based on the maximum likelihood estimates of the true positive and false negative of individual contours. It estimates the best agreement among individual contours and produces a consensus contour (reference contour) that best represents the underlying anatomy. Neither the expert contours nor the reference contour is displayed to the trainee any time. The trainee contour is then compared with the reference contour by using the LSSD metric to produce an LSSD map, which is then displayed to the user in the form of a 2D color map beside the contouring interface. The 2D LSSD maps are organized vertically by the CT slice, and horizontally by the sector angle, as illustrated in **Figure 2**. A positive LSSD indicates that the trainee contour is beyond the reference contour within that sector and slice, and a negative LSSD indicates that the trainee contour is within the reference contour in that sector and slice.

**Figure 3:**
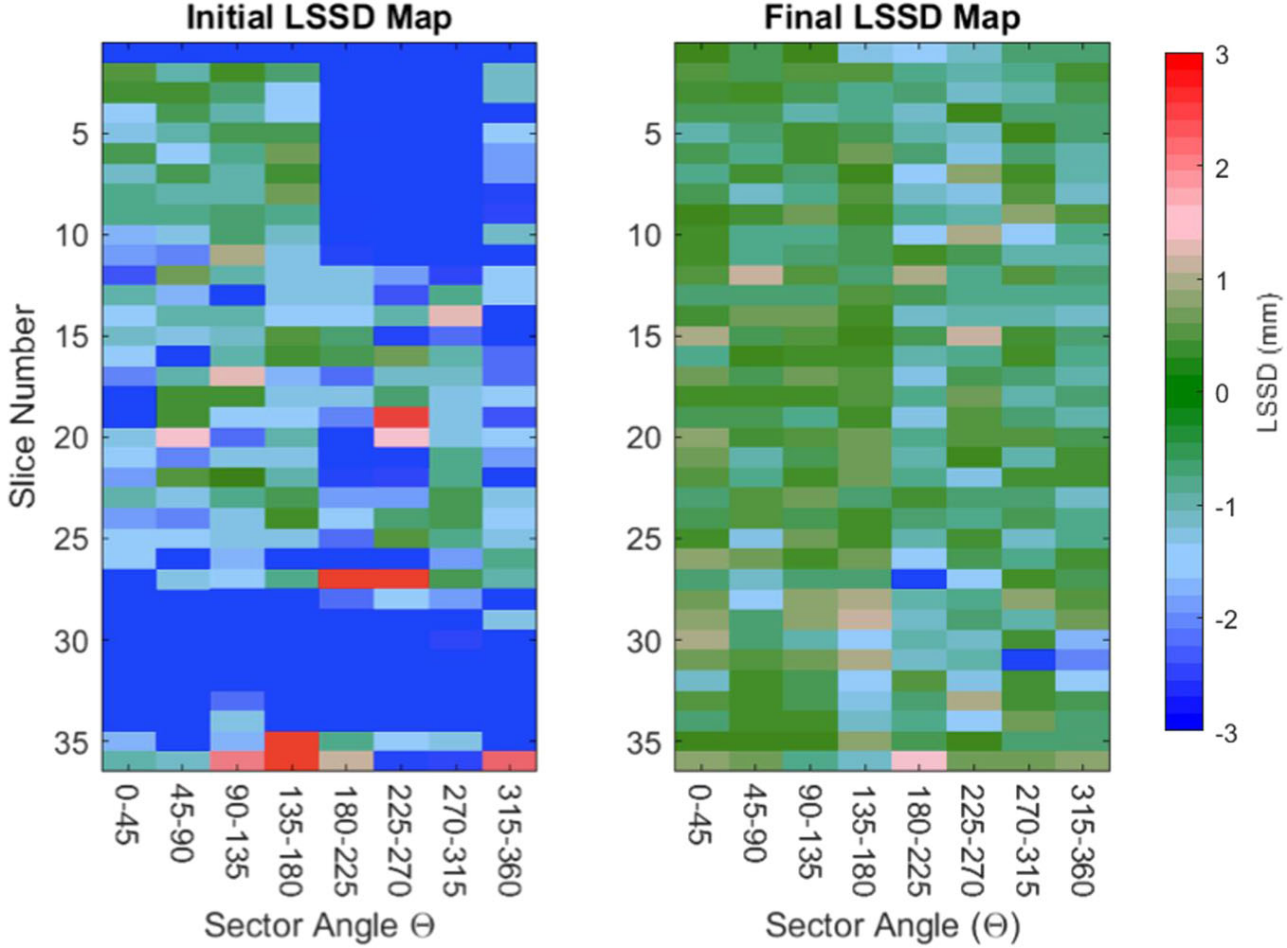
Representative initial and final LSSD maps for one trainee. Two LSSD colormaps are presented as the contourer progresses through a training session. The initial LSSD map (left) has large sections of red and blue, indicating that the trainee’s contour is more than 3 mm from the reference contour in that sector. The final LSSD map (right) has an overall color closer to green, indicating that the trainee’s contours are approaching an LSSD of 0.

After the trainee completes the initial contour, the LSSD map can be updated as often as needed while the contours are revised. The trainees were instructed to update the reference contour periodically during the contouring process. An updated reference contour (consensus contour) is recreated using the updated trainee contour and the stored expert contours through the STAPLE algorithm. The LSSD map is interactive in that one can select an LSSD unit, and the contouring interface will advance to the corresponding slice and highlight the sector of interest. As the trainee begins modifying their contours, the LSSD map is updated with the current value. LSSD values near zero are displayed as green; LSSD values of ≥ +3 mm are displayed as red; and LSSD values of ≤ –3 mm are displayed as blue. The LSSD map displayed to the trainee then saturates such that deviations in LSSD larger than 3 mm are displayed as red and blue. As the user adjusts their contour, the LSSD colormap is updated to indicate the trainee contour compared with the updated reference contour. Initial and final LSSD colormaps as the trainee progresses through contour training are shown in **Figure 3**. As the trainee progresses through training, the overall color of the LSSD map shifts towards green, i.e., an LSSD of zero.

### 2.5 Contour training sessions

Nine trainees were recruited for contour training using this software tool to evaluate the training process and its effectiveness. The trainees were medical physics staff with some knowledge of human anatomy but had not necessarily been trained in anatomy delineation from CT images. Each participating trainee was assigned six training cases, and one of those six cases was used for the retention case. The trainees were instructed to contour each case in an assigned order, which was chosen randomly to eliminate variation in contouring on a case-by-case basis. After each trainee had contoured the six training cases, the trainee waited 30 days to contour the retention case, to test whether the learned contouring skills were retained after a break in using the software. As noted, the retention case was the last case contoured in each trainee’s training session.

The LSSD maps were saved during the contour training sessions to quantify the effectiveness of the training. Each grid of the 2D LSSD map has a value representing the distance of the trainee contour from the reference contour within that particular slice and sector. A histogram of the LSSD values was generated for each LSSD map, and the average and standard deviation (LSSD_AVG_, LSSD_SD_) were used as metrics to quantify how the trainee’s present contour as a whole deviated from the reference contour. These values were computed for the initial and final LSSD maps (LSSD_AVG(i)_, LSSD_SD(i)_, LSSD_AVG(f)_ *,*LSSD_SD(F)_). For each trainee and each training case, the difference in LSSD_SD(i)_ and LSSD_SD(f)_ was computed to assess the functionality of the training tool (ΔLSSD_SD_). Statistical significance was computed by using a two-tailed *F* test at the 95% confidence level. After this, the initial LSSD maps of all trainees and all training cases were combined into a single data set to compare with the consolidated final LSSD maps. The same metrics were computed for the data sets to assess the overall performance of the training tool.

## 3 Results

**Figure 4:**
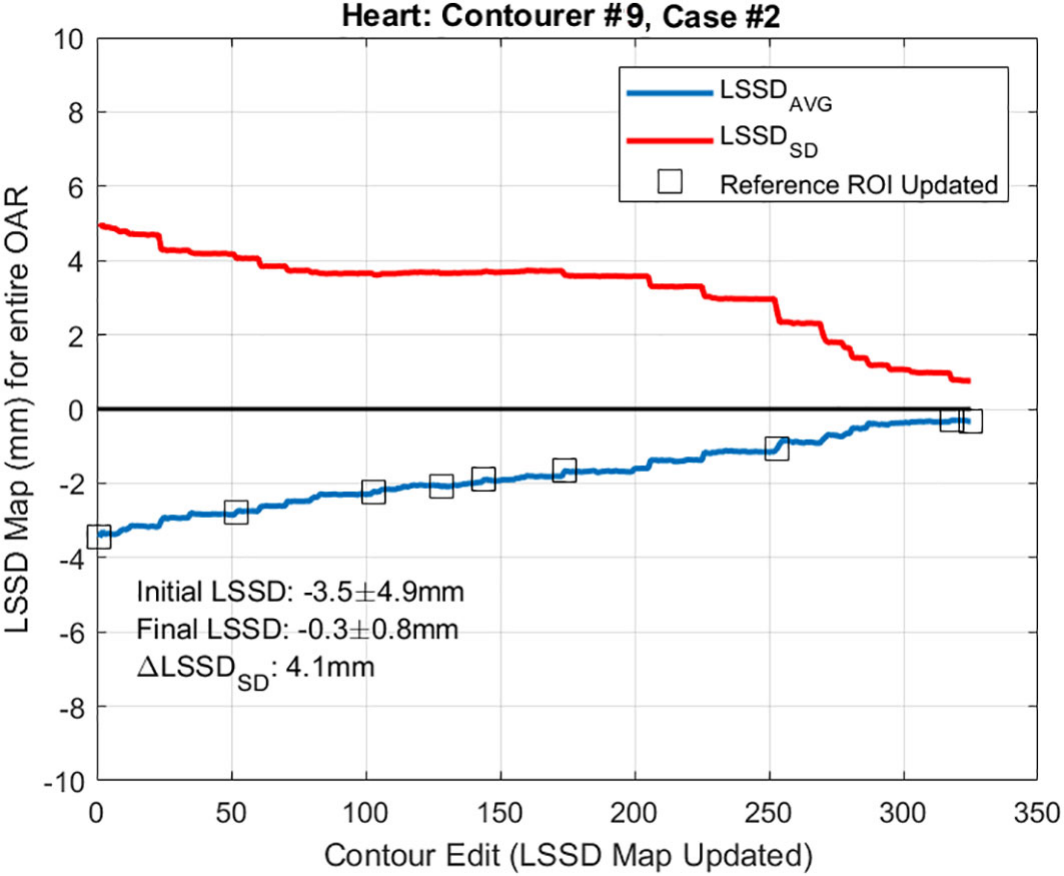
LSSD_AVG_ and LSSD_SD_ during contour training. Plot of the LSSD_AVG_ and LSSD_SD_ as trainee #9 progressed through the contour training process while contouring the heart. The x-axis on this graph represents each time the LSSD map was updated. ROI, region of interest; OAR, organ at risk.

The contour training tool was successfully developed, validated, and tested by nine different individuals to ensure proper function of the contouring interface. **Figure 4** shows a plot of the LSSD_AVG_ and LSSD_SD_ as a trainee progressed through contouring (the x-axis represents each time an LSSD map was regenerated). The graph of LSSD_SD_ represents each trainee’s progression during the contouring process, in that it trends towards 0 as the trainee uses the contour training tool to finalize their contours.

**Figure 5:**
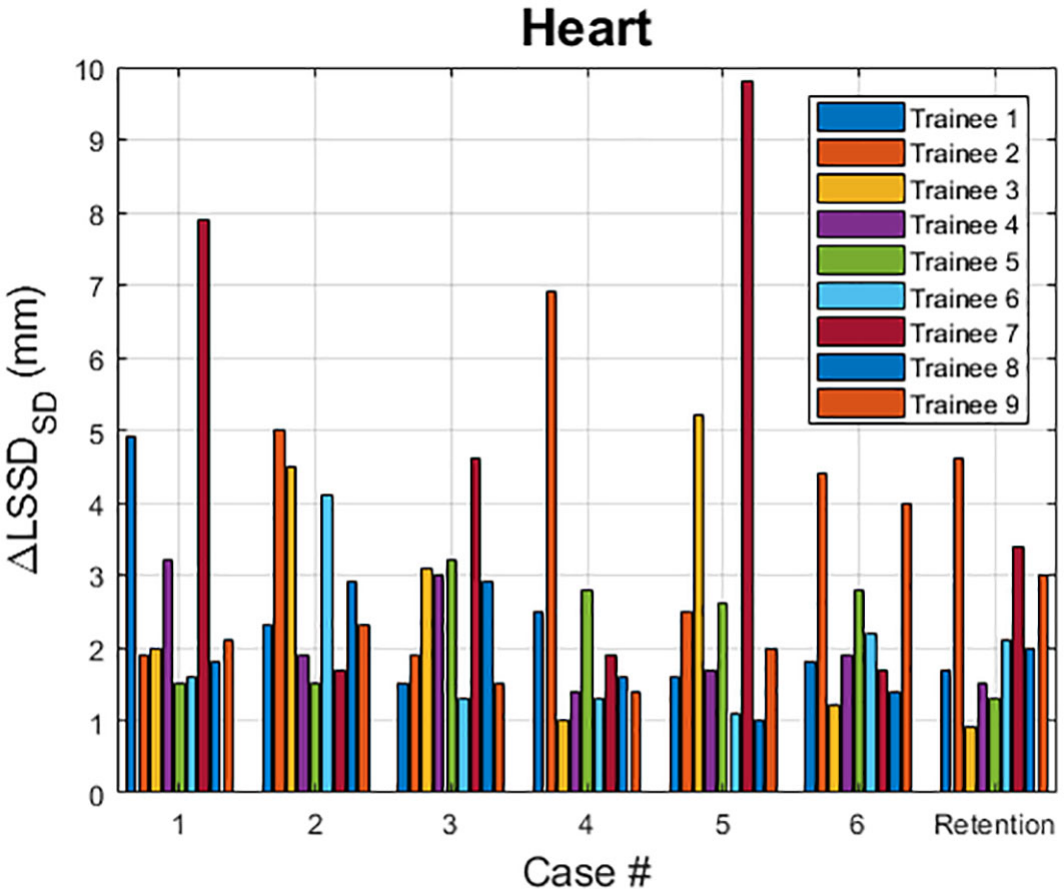
ΔLSSD_SD_ for the heart. ΔLSSD_SD_ values for the heart are plotted for each assigned training case as well as the retention case. For each case, all nine trainees’ results are displayed.

**Figure 6:**
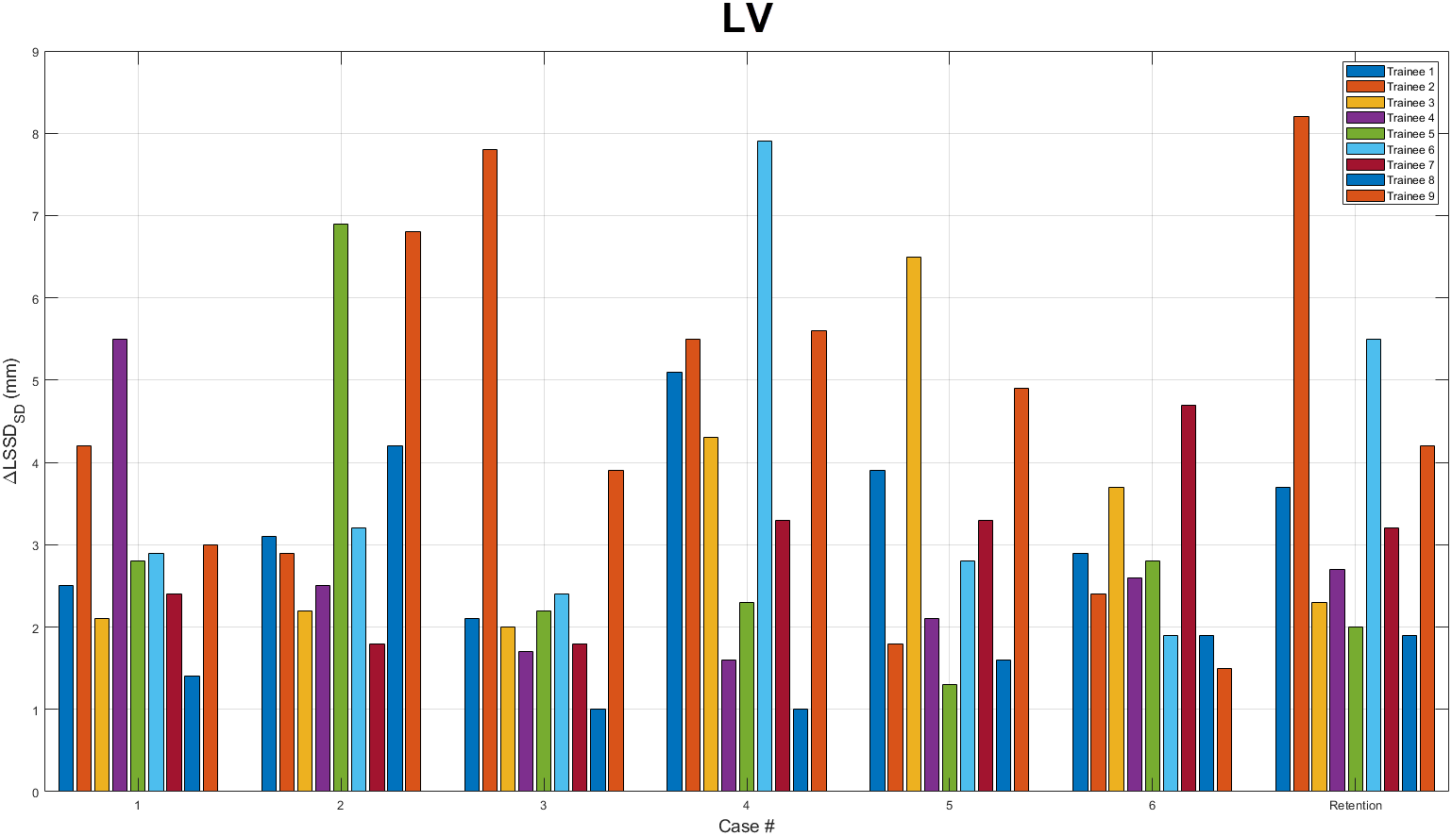
ΔLSSD_SD_ for the left ventricle. ΔLSSD_SD_ values for the left ventricle (LV) are plotted for each assigned training case as well as the retention case. For each case, all nine trainees’ results are displayed.

Next, the LSSD_AVG(i)_, LSSD_SD(i)_, LSSD_AVG(F)_ *,*LSSD_SD(F)_ were computed and tabulated for each trainee and each training case and are tabulated in **Table 1** for the heart and **Table 2** for the LV. For every case, including the retention cases, a statically significant ΔLSSD_SD_ was observed. The ΔLSSD_SD_ is plotted in **Figure 5** for the heart and in **Figure 6** for the LV for each trainee. For all trainees and all cases, the initial and final LSSD maps were aggregated and a ΔLSSD_SD_ was computed for both the training and retention cases. In that comparison, the ΔLSSD_SD_ for the heart was 3.4 mm for the training cases and 2.7 mm for the retention set. For the LV, the ΔLSSD_SD_ for the entire set was 4.1 mm for the training cases and 4.4 mm for the retention cases. These statistically significant ΔLSSD_SD_ values are a strong indication that the contour training tool aided the trainees in the contouring process so that their contours became more consistent with the reference expert contours after the training. No statistically significant differences in ΔLSSD_SD_ were observed between each trainee’s last training case and the retention cases.

**Table 1:** LSSD_AVG(i),_ (LSSD_SD(i)_), LSSD_AVG(F),_ (LSSD_SD(F)_) for contour training of the heart.

Trainee	Case 1	Case 2	Case 3	Case 4	Case 5	Case 6	Retention
1	4.5 (5.4), 0.2 (0.6)	0.2 (2.9), 0.0 (0.7)	–0.0 (2.1), 0.0 (0.7)	0.8 (3.1), –0.0 (0.6)	–0.2 (2.3), –0.0 (0.7)	0.8 (2.4), 0.0 (0.6)	–0.5 (2.3), –0.1 (0.5)
2	–1.2 (2.9), 0.1 (1.0)	–5.2 (5.8), –0.1 (0.8)	0.7 (2.6), 0.1 (0.7)	–5.2 (7.5), –0.1 (0.7)	–1.0 (3.2), –0.1 (0.7)	–2.5 (5.1), –0.1 (0.8)	–4.4 (5.3), –0.3 (0.8)
3	–3.3 (2.9), -0.4 (0.8)	–4.6 (5.4), –0.4 (0.9)	–0.5 (3.8), –0.2 (0.7)	–0.9 (1.7), –0.1 (0.8)	–1.3 (6.2), –0.1 (1.1)	–0.7 (1.9), –0.2 (0.7)	–1.1 (1.6), –0.1(0.7)
4	1.8 (4.1), 0.2 (0.9)	0.5 (2.8), 0.2 (0.8)	2.4 (3.8), 0.3 (0.7)	–1.0 (2.2), 0.1 (0.8)	–1.0 (2.4), 0.1 (0.7)	–0.0 (2.6), 0.1 (0.7)	–1.4 (2.3), –0.1 (0.7)
5	0.8 (2.2), 0.3 (0.7)	–0.7 (2.3), –0.1 (0.8)	1.1 (4.0), 0.0 (0.9)	–1.5 (3.6), –0.3 (0.8)	2.0 (3.3), 0.3 (0.7)	–0.8 (3.5), –0.1 (0.8)	–0.5 (2.1), -0.1 (0.8)
6	0.7 (2.5), 0.0 (0.8)	–3.5 (4.9), –0.3 (0.8)	–0.1 (2.1), 0.1 (0.8)	–0.1 (2.3), –0.1 (1.0)	–0.5 (2.0), –0.2 (0.9)	0.2 (3.4), –0.3 (1.2)	–0.7 (2.9), –0.4 (0.8)
7	–7.0 (8.6), –0.0 (0.7)	–1.7 (2.4), –0.3 (0.7)	–3.5 (5.3), –0.3 (0.7)	–1.6 (2.5), –0.6 (0.6)	–11.7 (10.5), –0.5 (0.7)	–1.4 (2.5), –0.2 (0.8)	–2.5 (4.0), –0.3 (0.6)
8	–1.7 (2.5), –0.4 (0.7)	–2.6 (3.7), –0.2 (0.8)	–0.9 (3.9), –0.3 (1.0)	0.5 (2.4), 0.0 (0.8)	–0.1 (2.6), –0.0 (1.6)	0.2 (2.1), 0.1 (0.7)	–0.7 (2.8), –0.0 (0.9)
9	0.7 (2.9), 0.1 (0.8)	0.5 (3.1), –0.0 (0.8)	1.4 (2.3), 0.1 (0.7)	–0.2 (2.2), 0.0 (0.8)	–2.0 (2.8), –0.0 (0.8)	–1.1 (4.7), 0.0 (0.7)	–2.1 (3.7), –0.2 (0.7)

**Table 2:** LSSD_AVG(i),_ (LSSD_SD(i)_), LSSD_AVG(F),_ (LSSD_SD(F)_) for contour training of the left ventricle.

Trainee	Case 1	Case 2	Case 3	Case 4	Case 5	Case 6	Retention
1	1.4 (3.0), 0.3 (0.5)	0.6 (4.3), 0.5 (1.2)	1.8 (2.7), 0.2 (0.5)	–1.4 (5.6), 0.1 (0.5)	1.8 (4.5), 0.3 (0.6)	0.9 (3.5), 0.1 (0.6)	1.4 (4.2), 0.4 (0.5)
2	–0.9 (5.0), 0.2 (0.8)	2.9 (3.6), 0.3 (0.7)	4.9 (8.4), 0.1 (0.6)	–3.4 (6.2), 0.0 (0.8)	0.5 (2.5), 0.2 (0.7)	–1.5 (3.1), –0.1 (0.7)	4.6 (8.8), 0.3 (0.6)
3	–1.0 (2.9), –0.0 (0.8)	–0.6 (2.9), 0.1 (0.7)	–2.1 (2.7), –0.0 (0.7)	–3.1 (5.0), 0.0 (0.7)	–2.8 (7.3), 0.0 (0.8)	–2.3 (4.4), 0.1 (0.7)	–0.9 (3.1), 0.2 (0.8)
4	–1.3 (6.3), 0.3 (0.7)	3.4 (3.2), 0.5 (0.7)	1.2 (2.4), 0.4 (0.6)	1.4 (2.2), 0.4 (0.7)	0.8 (2.6), 0.5 (0.6)	2.5 (3.2), 0.5 (0.6)	2.9 (3.3), 0.6 (0.6)
5	3.2 (3.6), 0.4 (0.7)	–0.2 (7.6), 0.2 (0.6)	–1.6 (2.9), 0.0 (0.7)	1.9 (2.9), 0.2 (0.6)	–0.4 (1.9), –0.0 (0.6)	1.9 (3.5), 0.2 (0.7)	1.9 (2.7), 0.2 (0.8)
6	–0.6 (4.2), –0.3 (1.3)	–0.5 (4.4), –0.2 (1.2)	–0.0 (3.3), –0.1 (1.0)	8.2 (8.7), 0.4 (0.9)	–1.5 (3.5), –0.1 (0.7)	0.5 (2.6), –0.0 (0.7)	4.1 (6.3), 0.0 (0.7)
7	1.2 (3.1), 0.2 (0.6)	0.9 (2.4), 0.1 (0.6)	0.1 (2.4), 0.2 (0.6)	–0.3 (4.0), 0.2 (0.7)	–2.1 (3.8), –0.1 (0.6)	–0.1 (5.3), 0.1 (0.6)	0.7 (3.8), 0.1 (0.6)
8	0.9 (2.2), 0.0 (0.8)	1.5 (4.9), 0.2 (0.7)	0.2 (1.8), 0.2 (0.8)	0.3 (1.7), 0.0 (0.7)	1.1 (2.4), 0.3 (0.7)	1.3 (2.7), 0.2 (0.8)	1.3 (2.7), 0.1 (0.8)
9	–1.6 (3.8), –0.1 (0.8)	3.5 (7.4), 0.2 (0.7)	–1.8 (4.6), 0.1 (0.7)	–2.7 (6.3), 0.1 (0.7)	–3.7 (5.5), –0.1 (0.6)	0.0 (2.0), 0.1 (0.5)	–0.5 (4.7), 0.1 (0.5)

## 4 Discussion

Variability is well known to exist in the contouring process and remains a challenge in radiotherapy (3, 5, 8). The variability not only results from different experience of clinicians and their training on how to generate consensus contours, but also can be significantly influenced by the image quality of the contouring dataset. Our previously has shown this variability in contouring cardiac substructures. The calcification, metal artifacts, and blurry from respiratory motion can all contribute to the contouring variability (17). Although that contour training can reduce this variability, currently available training methods or tools are either greatly time-consuming, lack real-time quantitative feedback, or are susceptible to variability among even experienced radiation oncologists who are providing the training. The contour training tool developed here has the capability to distribute expert contouring knowledge to a broad range of trainees with different backgrounds through established formal training sessions, so the trainees can improve their consensus contouring skill without the need for radiation oncology experts to be present. One substantial advantage of this tool is that it provides immediate feedback to the user as they contour a structure, which historically has been provided by a supervising radiation oncology staff member or by evaluating current anatomy on a CT scan against a reference, i.e., a peer-reviewed data set. We demonstrated the effectiveness of this tool by contouring the heart and LV; however, this tool can easily be extended to contour other organs or treatment targets. Expanding this tool to cover other anatomical sites such as head and neck is our future study. Indeed, our tool has enormous potential for reaching many end users who require a means of accurately delineating anatomic structures with limited training resources (including access to radiation oncologist experts). We expect that this tool will be particularly useful in low and middle-income countries where trained radiation oncology staff are needed but available resources are limited. In addition, nowadays autocontouring has become more and more popular and gradually replaces manual contouring in routine clinic. However, quality check of autocontours still relies on clinicians. Therefore, this tool is still be particularly useful to train clinical staff in identifying correct anatomical structures for autocontouring quality assurance.

Our tool creates the reference contour by fusing the trainee contour with expert contours by using the STAPLE algorithm. By doing so, we acknowledge that the ‘ground truth’ contour is unknown. The reference contour is the consensus contour contributed by both experts and the trainee. The STAPLE algorithm is based on the maximum likelihood estimates of the true positive and false negative of individual contours. If the trainee generates contours close to expert contours, a higher weight will be assigned to the trainee contour in generating the reference contour so that the reference contour could potentially favor the evaluation. On the other hand, if the trainee generates contours away from expert contours, the contribution to reference contour from the trainee contour will be small, which will unfavorable to the evaluation. This method has the potential to increase the sensitivity of consensus contour evaluation and also reduces the impact of inconsistent contours from experts to the evaluation.

To properly generate consensus contours (reference contours) by using the STAPLE algorithm, at least 3 expert contours are needed for each training structure. Also, to reduce the impact from trainee contour in generating reference contour, more expert contours are preferred. Therefore, expanding this software tool to cover training for other organs or treatment targets will require a significant effort to curate the expert contours. Establishing the gold standard via expert contours is the key to the use of this tool. A diverse group of expert contours needs to be evaluated. Also, the quality of the contour training depends on the quality of the expert contours, which can vary from physician to physician and across different institutions. This data curation process is often quite time-consuming. However, as more and more high-quality benchmark datasets become available, such as The Cancer Imaging Archive (26), we expect to be able to easily expand the usability of our contour training tool to include more training cases and structures.

One limitation of this software tool is that our new LSSD metric can only handle regular shape structures with the geometric center within the contour. Most normal organs have a regular shape in 2D slices, and their contouring can be trained using this tool. However, for some structures with a complicated shape, such as optic chiasm and brachial plexus, our software tool is not applicable. In addition, this tool works functions when contouring axial slices, however some contours are better generated in the sagittal and coronal planes. Further development of this utility would need to accommodate for contouring on non-axial reconstructed planes.

## 5 Conclusion

A software utility that served as a contour training tool was developed, tested, and implemented. This tool allowed users to be trained on the contouring process with real-time feedback on their contouring performance in terms of consistency with multiple contours by expert radiation oncologists. The software was designed with flexibility in mind so that it can be used to contour any anatomic site. For all cases tested, the trainees were able to use the training software to modify their contours to be more consistent with those of the experts. Although this study was done as a proof of principle, the software could easily be implemented on a larger scale for radiation oncology residents, junior faculty, and even senior faculty who need a refresher course on contour training. This tool could also be used for training dosimetrists and therapy staff who wish to improve both their knowledge of and consistency in anatomic contouring.

## Data availability statement

The original contributions presented in the study are included in the article/supplementary material. Further inquiries can be directed to the corresponding authors.

## Ethics statement

The studies involving humans were approved by The University of Texas MD Anderson Cancer Center. The studies were conducted in accordance with the local legislation and institutional requirements. The ethics committee/institutional review board waived the requirement of written informed consent for participation from the participants or the participants’ legal guardians/next of kin because this is a retrospective study and only retrospective image data are used in this study.

## Author contributions

CLN, LC, MM, and JY contributed to conception and design of the study. CLN, DR, TN, RM, SG, CG, BM, MEB, YZ, MG, SH, and KN collected the contouring data. CLN and CC performed data analysis. CN, RF, and JY developed the contouring training software. LC and MM secured funding for this study. CLN and JY wrote the first draft of the manuscript. All authors contributed to the article and approved the submitted version.

## References

[1] Ezzell GA Galvin JM Low D Palta JR Rosen I Sharpe MB. Guidance document on delivery, treatment planning, and clinical implementation of IMRT: Report of the IMRT subcommittee of the AAPM radiation therapy committee. Med Phys (2003) 30(8):2089–115. doi: 10.1118/1.1591194 12945975

[2] Mackie TR Kapatoes J Ruchala K Lu W Wu C Olivera G. Image guidance for precise conformal radiotherapy. Int J Radiat Oncol Biol Physics. (2003) 56(1):89–105. doi: 10.1016/s0360-3016(03)00090-7 12694827

[3] Li XA Tai A Arthur DW Buchholz TA Macdonald S Marks LB. Variability of target and normal structure delineation for breast cancer radiotherapy: An RTOG Multi-Institutional and Multiobserver Study. Int J Radiat Oncol Biol Phys (2009) 73(3):944–51. doi: 10.1016/j.ijrobp.2008.10.034 PMC2911777 19215827

[4] Yang J Woodward WA Reed VK Strom EA Perkins GH Tereffe W. Statistical modeling approach to quantitative analysis of interobserver variability in breast contouring. Int J Radiat Oncol Biol Phys (2014) 89(1):214–21. doi: 10.1016/j.ijrobp.2014.01.010 PMC3997068 24613812

[5] Joskowicz L Cohen D Caplan N Sosna J. Inter-observer variability of manual contour delineation of structures in CT. Eur Radiol (2019) 29(3):1391–9. doi: 10.1007/s00330-018-5695-5 30194472

[6] Sharp G Fritscher KD Pekar V Peroni M Shusharina N Veeraraghavan H. Vision 20/20: Perspectives on automated image segmentation for radiotherapy. Med Phys (2014) 41(5):050902. doi: 10.1118/1.4871620 24784366 PMC4000389

[7] Owens CA Peterson CB Tang C Koay EJ Yu W Mackin DS. Lung tumor segmentation methods: Impact on the uncertainty of radiomics features for non-small cell lung cancer. PLoS One (2018) 13(10):e0205003. doi: 10.1371/journal.pone.0205003 30286184 PMC6171919

[8] Louie AV Rodrigues G Olsthoorn J Palma D Yu E Yaremko B. Inter-observer and intra-observer reliability for lung cancer target volume delineation in the 4D-CT era. Radiother Oncol (2010) 95(2):166–71. doi: 10.1016/j.radonc.2009.12.028 20122749

[9] Bradley JD Paulus R Komaki R Masters G Blumenschein G Schild S. Standard-dose versus high-dose conformal radiotherapy with concurrent and consolidation carboplatin plus paclitaxel with or without cetuximab for patients with stage IIIA or IIIB non-small-cell lung cancer (RTOG 0617): A randomised, two-by-two factorial phase 3 study. Lancet Oncol (2015) 16(2):187–99. doi: 10.1016/S1470-2045(14)71207-0 PMC4419359 25601342

[10] eContour. Available at: https://econtour.org/ (Accessed 2021 November 18).

[11] EduCase. Available at: https://www.educase.com/ (Accessed 2021 November 18).

[12] Datta NR Samiei M Bodis S. Radiation therapy infrastructure and human resources in low- and middle-income countries: Present status and projections for 2020. Int J Radiat Oncol Biol Phys (2014) 89(3):448–57. doi: 10.1016/j.ijrobp.2014.03.002 24751411

[13] McCarroll R Beadle B Balter P Burger H Cardenas C Dalvie S. Retrospective validation and clinical implementation of automated contouring of organs at risk in the head and neck: A step toward automated radiation treatment planning for low- and middle-income countries. J Global Oncol (2018) 4:1–11. doi: 10.1200/Jgo.18.00055 PMC6223488 30110221

[14] Karim S Sunderji Z Jalink M Mohamed S Mallick I Msadabwe-Chikuni SC. Oncology training and education initiatives in low and middle income countries: A scoping review. Ecancermedicalscience (2021) 15:1296. doi: 10.3332/ecancer.2021.1296 34824619 PMC8580602

[15] Rogelj P Hudej R Petric P. Distance deviation measure of contouring variability. Radiol Oncol (2013) 47(1):86–96. doi: 10.2478/raon-2013-0005 23450669 PMC3573839

[16] Gros SAA Xu W Roeske JC Choi M Emami B Surucu M. A novel surrogate to identify anatomical changes during radiotherapy of head and neck cancer patients. Med Phys (2017) 44(3):924–34. doi: 10.1002/mp.12067 28019647

[17] Zhou R Liao Z Pan T Milgrom SA Pinnix CC Shi A. Cardiac atlas development and validation for automatic segmentation of cardiac substructures. Radiother Oncol (2017) 122(1):66–71. doi: 10.1016/j.radonc.2016.11.016 27939201 PMC5292289

[18] Niedzielski JS Wei X Xu T Gomez DR Liao Z Bankson JA. Development and application of an elastic net logistic regression model to investigate the impact of cardiac substructure dose on radiation-induced pericardial effusion in patients with NSCLC. Acta Oncol (2020) 59(10):1193–200. doi: 10.1080/0284186X.2020.1794034 32678696

[19] Sardaro A Petruzzelli MF D'Errico MP Grimaldi L Pili G Portaluri M. Radiation-induced cardiac damage in early left breast cancer patients: Risk factors, biological mechanisms, radiobiology, and dosimetric constraints. Radiother Oncol (2012) 103(2):133–42. doi: 10.1016/j.radonc.2012.02.008 22391054

[20] Aleman BMP Moser EC Nuver J Suter TM Maraldo MV Specht L. Cardiovascular disease after cancer therapy. Eur J Cancer Suppl (2014) 12(1):18–28. doi: 10.1016/j.ejcsup.2014.03.002 PMC4250533 26217163

[21] Luo Y Xu Y Liao Z Gomez D Wang J Jiang W. Automatic segmentation of cardiac substructures from noncontrast CT images: Accurate enough for dosimetric analysis? Acta Oncol (2019) 58(1):81–7. doi: 10.1080/0284186X.2018.1521985 PMC6377299 30306817

[22] RTOG. Atlases for Organs at Risk (OARs) in Thoracic Radiation Therapy . Available at: https://www.rtog.org/CoreLab/ContouringAtlases/LungAtlas.aspx (Accessed 2019 March 08).

[23] Feng M Moran JM Koelling T Chughtai A Chan JL Freedman L. Development and validation of a heart atlas to study cardiac exposure to radiation following treatment for breast cancer. Int J Radiat Oncol Biol Phys (2011) 79(1):10–8. doi: 10.1016/j.ijrobp.2009.10.058 PMC2937165 20421148

[24] Warfield SK Zou KH Wells WM. Simultaneous truth and performance level estimation (STAPLE): An algorithm for the validation of image segmentation. IEEE Trans Med Imaging (2004) 23(7):903–21. doi: 10.1109/TMI.2004.828354 PMC1283110 15250643

[25] Yang J Wei C Zhang L Zhang Y Blum RS Dong L. A statistical modeling approach for evaluating auto-segmentation methods for image-guided radiotherapy. Comput Med Imaging Graph (2012) 36(6):492–500. doi: 10.1016/j.compmedimag.2012.05.001 22673541 PMC3398201

[26] Clark K Vendt B Smith K Freymann J Kirby J Koppel P. Cancer imaging archive (TCIA): Maintaining and operating a public information repository. J Digit Imaging (2013) 26(6):1045–57. doi: 10.1007/s10278-013-9622-7 PMC3824915 23884657

